# Editorial: Toxicity Mechanisms, Exposure, Toxicokinetic and Risk Assessment Aspects of Metals, Toxic for Animals and Humans

**DOI:** 10.3389/fphar.2022.847028

**Published:** 2022-02-14

**Authors:** Yanzhu Zhu, Fatma M. El-Demerdash, Alex Boye, Michel Mansur Machado, Xinwei Li

**Affiliations:** ^1^ Institute of Special Animal and Plant Sciences of Chinese Academy Agricultural Science, Changchun, China; ^2^ Environmental Studies Department, Institute of Graduate Studies and Research, Alexandria University, Alexandria, Egypt; ^3^ Department of Medical Laboratory Science, School of Allied Health Sciences, College of Health and Allied Sciences, University of Cape Coast, Cape Coast, Ghana; ^4^ Universidade Federal do Pampa—Campus Uruguaiana, Uruguaiana, Brazil; ^5^ Key Laboratory of Zoonosis, Ministry of Education, College of Veterinary Medicine, Jilin University, Changchun, China

**Keywords:** toxicity mechanisms, toxicity exposure, toxicokinetic, risk assessment, metals

Toxicology is a translational science, transferring knowledge from basic science into practical applications to safeguard human health and the environment, thus evaluating dose-effect relationships from safe doses to doses eliciting adverse effects and the underlying mechanism responsible for the adverse effects. Globally, exposures to toxic metals through water, food, and the environment pose a major health threat to humans and animals. In particular, environmental and occupational metals exposure underlie many human and animal diseases. More needs to be done to address the major health concerns associated with heavy metal exposures. The present special topic received 20 articles highlighting toxicity of various metals/metalloids, their toxicity, mechanism, and promising therapeutic agents including anti-oxidants.

The work of Wang et al. finds that swainsonine (SW), an indolizidine alkaloid extracted from locoweeds, induces cell paraptosis through ER stress and MAPK signaling pathway, thus further laying a theoretical foundation for the study of SW toxicity mechanism. Guo et al. indicate that demonstration of ER stress is involved in the hepatotoxicity induced by monocrotaline (MCT), and CHOP plays an important role in this process. Alehaideb et al. found that furanocoumarin bioactive metabolism in humans would result in reactive metabolite(s) formation inactivating CYP1A2 isozyme and inhibiting caffeine metabolism. Once the CYP1A2 isozyme is deactivated, the enzymic activity can only be regained by isozyme re-synthesis which takes a long time. Deore et al. find that alpha-lipoic acid protects experimental rats against lead and ZnO NP-induced altered immunological, neurological, and reproductive characteristics. Alpha-lipoic acid’s therapeutic impact can be due to its multiple actions, which include chelation, antioxidative, anti-inflammatory, and antiapoptotic effects. Kim et al. studies skin sensitization by NPs using two skin sensitization test methods and find that CuO and CoO NPs have sensitization potential. The effect is induced by the constituent elements of fast-dissolving NPs. Metal ion release is verified as the key “factor” for cutaneous sensitivity based on ion chelation studies. Ta et al. evaluated a variety of skin sensitization predictive models that can be employed in the biopharmaceuticals and cosmeceuticals industries, as well as their future prospects and highlighted problems. *In vitro* skin sensitization tests alone cannot replace human and animal tests because they only focus on one single pathway in adverse outcome pathways (AOP). In silico approach, conversely, has more advantages than *in vitro* tests since it can take into account more than one AOP key event by combining various in chemico, *in vitro*, and *in vivo*. Sui et al. discovered that mogroside V (MV), a significant bioactive component of *S. grosvenorii*, may reduce oocyte meiotic abnormalities and quality degeneration in benzo 1) pyrene (BaP)-exposed mice by scavenging reactive oxygen species. According to Nogueira et al., the results of the transcriptome profile, together with their prior investigation, demonstrate physiological and genomic abnormalities produced by MeHg nonlethal concentration in a human salivary gland cell line. Despite being non-lethal, this concentration can induce oxidative stress, which causes changes in gene pathways associated with DNA integrity and biochemical reactions, both directly and indirectly. In their review, Balali-Mood et al. explore the harmful mechanisms of five heavy metals: mercury, lead, chromium, cadmium, and arsenic. ROS formation, antioxidant defense weakness, enzyme inactivation, and oxidative stress are all mechanisms that they share. These metals also interfere with biological functions such as growth, proliferation, differentiation, damage-repair, and apoptosis. Zhang et al. illustrated the metabolic alterations in Realgar-induced nephrotoxic mice through a metabolomic approach. Guo et al. find that the ethanol extraction process could induce severe PF hepatotoxicity. Bavachin, psoralidin, bavachinin, neobavaisoflavone, and bakuchiol are the main hepatotoxic ingredients. This mechanism could be associated with oxidative stress and mitochondrial damage mediated apoptosis. Zhang et al. found that overall, the present research reveals the possible underlying mechanisms by which BPFL exposure induced impairments and CA supplementation protected against these impairments in porcine SCs. Yang et al. demonstrate that three miRNAs, i.e., hsa-miR-148a-3p, hsa-miR-362-5p, and hsa-miR-194-5p, might serve as potential biomarkers for pyrrolizidine alkaloids-induced hepatic sinusoidal obstruction syndrome in clinics. Zou et al. find that inhibiting gap junction intercellular communication (GJIC) could delay the cytotoxic damage of cadmium and induce autophagy, but further block autophagic flux, promoting GJIC to obtain the opposite results.

In this special issue, knowledge and understanding of metals/metalloids with respect to their exposure dynamics, toxicokinetics, and mechanism of toxicity in humans and animals are critical for developing appropriate strategies to prevent, treat and manage metal/metalloid poisoning.

